# Semi-automated EEG Enhancement Improves Localization of Ictal Onset Zone With EEG-Correlated fMRI

**DOI:** 10.3389/fneur.2019.00805

**Published:** 2019-08-02

**Authors:** Simon Van Eyndhoven, Borbála Hunyadi, Patrick Dupont, Wim Van Paesschen, Sabine Van Huffel

**Affiliations:** ^1^Department of Electrical Engineering (ESAT), STADIUS Center for Dynamical Systems, Signal Processing and Data Analytics, Leuven, Belgium; ^2^Department of Microelectronics, TUDelft, Delft, Netherlands; ^3^Laboratory for Cognitive Neurology, Department of Neurosciences, KU Leuven, Leuven, Belgium; ^4^Leuven Brain Institute, Leuven, Belgium; ^5^Laboratory for Epilepsy Research, KU Leuven, Leuven, Belgium; ^6^Department of Neurology, University Hospitals Leuven, Leuven, Belgium

**Keywords:** EEG-fMRI, EEG-informed fMRI, signal enhancement, multi-channel Wiener filter, ictal onset zone, epilepsy, interictal epileptic discharge, presurgical evaluation

## Abstract

**Objective:** To improve the accuracy of detecting the ictal onset zone, we propose to enhance the epilepsy-related activity present in the EEG signals, before mapping their BOLD correlates through EEG-correlated fMRI analysis.

**Methods:** Based solely on a segmentation of interictal epileptic discharges (IEDs) on the EEG, we train multi-channel Wiener filters (MWF) which enhance IED-like waveforms, and suppress background activity and noisy influences. Subsequently, we use EEG-correlated fMRI to find the brain regions in which the BOLD signal fluctuation corresponds to the filtered signals' time-varying power (after convolving with the hemodynamic response function), and validate the identified regions by quantitatively comparing them to ground-truth maps of the (resected or hypothesized) ictal onset zone. We validate the performance of this novel predictor vs. that of commonly used unitary or power-weighted predictors and a recently introduced connectivity-based metric, on a cohort of 12 patients with refractory epilepsy.

**Results:** The novel predictor, derived from the filtered EEG signals, allowed the detection of the ictal onset zone in a larger percentage of epileptic patients (92% vs. at most 83% for the other predictors), and with higher statistical significance, compared to existing predictors. At the same time, the new method maintains maximal specificity by not producing false positive activations in healthy controls.

**Significance:** The findings of this study advocate for the use of the MWF to maximize the signal-to-noise ratio of IED-like events in the interictal EEG, and subsequently use time-varying power as a sensitive predictor of the BOLD signal, to localize the ictal onset zone.

## 1. Introduction

Epilepsy is one of the most common neurological disorders, affecting an estimated 50 million people worldwide (1). Of this group, about 30% of patients suffers from refractory epilepsy, characterized by recurrent seizures that cannot sufficiently be suppressed by anti-epileptic drugs. Hence, these patients are candidates to undergo resective surgery, in which the epileptogenic zone is removed or disconnected from surrounding tissue, with the goal of preventing further seizure generation. However, the epileptogenic zone is a mere abstract concept, defined as the “brain tissue whose removal/resection renders a patient seizure-free,” and there exists no method that measures it Rosenow and Luders (2). Hence, during the presurgical planning, different imaging modalities provide incomplete, but complementary clues on its exact location and should be consulted by neurologists to detect and segment the target for surgery. In this paper, we focus on the simultaneous recording of electroencephalography (EEG) and functional magnetic resonance imaging (fMRI), which has been an ubiquitous technique in brain research, and epilepsy research in particular (3–5).

The rationale behind simultaneously recording EEG-fMRI is the excellent temporal resolution of EEG (measuring neurophysiological changes on the order of milliseconds) and the fine spatial resolution of blood oxygen level dependent (BOLD) signals measured in fMRI (representing tissue oxygenation changes in local volumes of a few millimeter in diameter). Neuronal firing consumes energy and thus induces metabolic demands, which must be accommodated by an increased flow of oxygenated blood to the neural tissue. The temporal occurrence of such neural events can be tracked well using EEG, and by correlating these occurrences to the BOLD signals recorded at every voxel, it is possible to infer which brain regions participate in the generation or propagation of such events (6–8). In the context of refractory epilepsy, the events of interest are mostly interictal epileptic discharges (IEDs), as a surrogate of ictal periods (seizures), since they are subclinical and as such do not lead to e.g., movement-related artifacts in the EEG or fMRI data, as most types of seizures do (2). Hence, this approach crucially relies on the assumption that the regions involved in interictal activity (named the irritative zone) coincide, or at least overlap, with the ictal onset zone (IOZ), which is the actual target for resective surgery. We have studied this hypothesis in Tousseyn et al. (9), where it was demonstrated that ictal networks correspond well to interictal networks, by comparing images from single-photon emission computed tomography (SPECT) and fMRI. Beyond the similarity at the network level, also the site of onset of seizures and IEDs shows remarkable correspondence, based on evidence from intracranial EEG recordings (10). The role of IEDs as precursors of seizures, by strengthening or sustaining specific axonal connections that make up the epileptic network, has been discussed in Rosenow and Luders (2) and Staley and Dudek (11). Furthermore, there is evidence that the highest BOLD activations associated to IEDs, do indeed coincide with the seizure onset zone (12, 13). Backed up by this body of evidence, we may expect that IEDs provide a valid alternative to seizures when it comes to mapping of the epileptogenic zone.

The irritative zone or network can then be mapped by means of EEG-correlated fMRI analysis, as explained before. In this workflow, a time-varying representation of the IEDs is constructed, which is used to predict the BOLD time series through a model of the hemodynamic response. Voxels where the predicted time series correlates well to the measured BOLD time series are then considered to be part of the irritative network (6). The success of this approach hence depends on the accurate modeling of the neurovascular coupling and the timing of the IEDs. Many heuristic IED representations have been devised, to capture electrophysiological features that are suited to reveal concomitant BOLD changes. Many of these heuristic predictors rely on the manual delineation of IEDs by a neurologist, which is time-consuming and subjective. Recently, EEG synchronization measures were proposed in Abreu et al. (14) to construct a predictor time course, based on the observation that epileptic activity is marked by excessive neuronal synchronization. The authors argue that existing predictors were mostly based on the amplitude of the EEG signals [see e.g., (7, 8)], and as such are susceptible to artifacts and noise, and ignore important connectivity aspects in the EEG data.

In this paper we propose another paradigm to find predictors that are as informative as possible. Our approach is based on the enhancement of IED-like activity in the EEG signals, while suppressing irrelevant background activity, noise, and artifacts as much as possible. Specifically, we employ a multi-channel Wiener filter as in Somers et al. (15) to maximize this contrast, after which we derive the predictor as the power in the filtered EEG signals. The goal of this paper is to evaluate the feasibility of this approach, and compare its performance to that of several other existing predictors, based on stick models of the IED (3), on IED amplitude (16), global field synchronization (17), and the phase slope index (18). To investigate the viability of simultaneous EEG-fMRI recording as one of the presurgical mapping techniques, it is important to assess performance by means of a very direct, clinical metric (5, 19, 20). Here, we accomplish this by studying a cohort of refractory focal epilepsy patients for which ground truth information on the IOZ is available, which we described earlier in Tousseyn et al. (21).

## 2. Methods and Materials

### 2.1. Patient Group

We used data of twelve patients with both left- and right-hemispheral refractory focal epilepsy, whom we previously studied in Tousseyn et al. (9, 21–23). These patients were selected based on the following criteria (21): (1) adults who underwent a full presurgical evaluation for refractory focal epilepsy, including seizure history, neurological and physical examination, neuropsychological assessment, interictal and ictal scalp EEG recordings, video analysis of seizures, high-resolution MRI of the brain, and in most patients SISCOM and interictal ^18^F-FDG PET. In selected cases, intracranial EEG-recordings were performed; (2) concordant data pointing to one epileptic focus using all available presurgical investigations (including a SISCOM) *or else* successful outcome after epilepsy surgery [international league against epilepsy (ILAE) outcome classification 1–3 (1, completely seizure-free; 2, only auras; 3, one to three seizure days per year ± auras; 4, four seizure days per year to 50% reduction of baseline seizure days ± auras; 5, <50% reduction of baseline seizure days to 100% increase of baseline seizure days ± auras; 6, more than 100% increase of baseline seizure days ± auras)]; (3) recording of interictal spikes during EEG-fMRI. Nine patients included in the study had some form of temporal lobe epilepsy (TLE). We present an overview of the clinical data of all included patients in Table 1. Note that patients 2 and 6 had a poor outcome after surgery (ILAE classification 4 and 5, respectively), which was likely the result of incomplete surgery, constrained by the cortical location of the IOZ. In the former case, part of the dysplastic lesion was located in the motor cortex, and was not resected. In the latter case, the dysplastic lesion was located in the primary motor cortex, and the surgeon did not remove all cortical tissue constituting the lesion. Therefore, an investigation of the concordance of EEG-fMRI with the other modalities' evidence is still considered meaningful in these cases. To be able to assess the specificity of the evaluated methods [as in (21)], we incorporated fMRI recordings of twelve healthy controls, which were age- and gender-matched to the patients. We discuss the importance of the control group in more detail in section 2.6. This study was carried out in accordance with the recommendations of the International Conference on Harmonization guidelines on Good Clinical Practice with written informed consent from all subjects. All subjects gave written informed consent in accordance with the Declaration of Helsinki. The protocol was approved by the Medical Ethics Committee of the University Hospitals KU Leuven.

**Table 1:** Clinical patient data.

**Patient**	**Gender**	**Ictal onset zone**	**Etiology**	**Surgery**	**ILAE outcome**	**Follow-up time after surgery (y)**
1	F	L temporal	HS	Temporal lobe resection	3	5
2	F	L parietal	FCD	Partial lesionectomy	4	5
3	F	R parieto- occipito-temporal	Sturge-Weber			
4	M	R temporal	Unknown			
5	F	L anterior Temporal	HS	Temporal lobe resection	1	8
6	F	R frontal	FCD	Partial lesionectomy	5	2
7	F	L anterior temporal	DNET	Temporal lobe resection	1	4
8	M	L temporo- parietal	unknown	Overlap eloquent cx		
9	F	L occipital	FCD	Overlap eloquent cx		
10	F	R temporal	HS	Refused		
11	M	L anterior temporal	HS	Temporal lobe resection	1	6
12	F	R temporal	CNS infection	Refused		

*F, female; M, male; L, left; R, right; CNS, central nervous system; DNET, dysembryoplastic neuroepihelial tumor; FCD, focal cortical dysplasia; HS, hippocampal sclerosis; cx, cortex*.

### 2.2. Data Acquisition and Preprocessing

Functional MRI data were acquired on one of two 3T MR scanners (Achieva TX with a 32-channel head coil and Intera Achieva with an eight-channel head coil, Philips Medical Systems, Best, The Netherlands) with an echo time (TE) of 33 ms, a repetition time (TR) of either 2.2 or 2.5 s, and a voxel size of 2.6 × 3 × 2.6 mm^3^. EEG data were recorded using MR-compatible caps with 30–64 electrodes, sampled at 5 kHz. The fMRI images were realigned, slice-time corrected, and normalized to MNI space, resampled to a voxel size of 2 × 2 × 2 mm^3^, and smoothed using a Gaussian kernel of 6 mm full width at half maximum (FWHM). All fMRI processing steps were carried out using SPM8 in Matlab 2015a. The EEG signals were band-pass filtered offline between 1 and 50 Hz, gradient artifacts were removed and pulse artifacts were subtracted, and the signals were downsampled to 250 Hz. We refer the reader to Tousseyn et al. (21) for a detailed description of all preprocessing steps. Two neurologists subsequently inspected and annotated the EEG signals for IEDs. Table 2 presents the number of annotated IEDs and the duration of the fMRI recording for every subject, along with the number of degrees of freedom for the *T*-tests that are carried out to map the interictal discharges (see section 2.4).

**Table 2:** List of the number of IEDs that were observed during the fMRI recording of every patient, as well as the degrees of freedom of the fMRI design matrix.

**Patient**	**Number of fMRI scans**	**# IEDs during fMRI recording**	**IED rate (# IEDs per hour) during fMRI**	**Degrees of freedom (DoF) of fMRI design matrix**
1	540	15	40	509
2	1620	663	589	1512
3	1080	105	156	1008
4	1620	825	733	1527
5	1080	117	156	1015
6	1080	640	853	1009
7	1080	126	187	1012
8	1080	11	15	1008
9	1620	1815	1613	1530
10	540	226	602	509
11	1080	6	8	1018
12	1350	966	1171	1265

### 2.3. EEG-Derived BOLD Predictors

In this paper, we aim to improve the model of interictal electrophysiological variations which are neurovascularly coupled to BOLD fluctuations. To this end, we focus on the derivation of suitable IED features from the EEG, which serve as *predictors* for the simultaneously recorded BOLD time series, after convolution with a hemodynamic response function (see section 2.4). To model the EEG-based IED correlate, several time-varying representations have been conceived and used in prior studies (7, 8, 14, 16). We discuss some of the most significant heuristics below, without attempting to be exhaustive. It is important to remark that all of the mentioned approaches, besides the Global Field Synchronization (GFS) predictor, in one way or another, require the prior labeling of IEDs on the EEG (either by a neurologist or by an automatic detection algorithm). In our study, all IEDs were manually annotated by a neurologist, and discussed with a second neurologist.

#### 2.3.1. Unitary Predictors (Un)

The simplest predictor is constructed by creating a time course with a stick function of unit amplitude at the time of every detected IED, and zeros everywhere else (3, 19). This “unitary” (Un) representation treats every IED occurrence in the same way, i.e., neglects differences in amplitude or duration (5).

#### 2.3.2. Time-Varying Power of Independent Components (ICApow)

As a first logical alternative to the discrete unitary predictors, power-based predictors have been proposed. Different varieties exist, but they all share the continuous aspect since they are computed from the time-varying power of the EEG signal. We employ the approach used in Abreu et al. (14) to allow fair comparison. First, independent component analysis (ICA) is performed on the *M*-channel EEG data **x**[*t*] ∈ ℝ ^*M*^ indexed by time *t*:

(1) $$x[t]=\hat{A}\hat{s}[t]$$

(2) $$\hat{s}[t]={\hat{A}}^{-1}x[t]$$

(3) $$≜{\hat{W}}^{T}x[t]$$

where $\hat{\text{A}}\in {\mathbb{R}}^{M\times M}$ is an estimated mixing matrix, describing the contribution of every estimated source or independent component (IC) in $\hat{\text{s}}\left[t\right]\in {\mathbb{R}}^{M}$ to every channel of **x**. Conversely, the estimated sources can be written as the output of *M* spatial filters in the columns of an unmixing matrix $\hat{\text{W}}$, applied to the data. From the *M* ICs, the one which is most related to the IEDs is then kept, according to the PROJIC procedure in Abreu et al. (24), which we summarize here. The EEG data epochs around the times of user-annotated IEDs are averaged, leading to a template IED over *M* channels and *T*_*w*_ time points, represented in a matrix $\text{Z}\in {\mathbb{R}}^{M\times T_{w}}$. Subsequently, the average IED is projected onto the IC space as $\text{P}={\hat{\text{W}}}^{T}\text{Z}$. Under the assumption that IED-related ICs attain a higher power in **P** than other, non-IED-related ICs, we can then select the IC which contributes the most signal power to the average IED, computed as the sum of squares of every row of **P**. This IC is then considered an estimated time course of interictal activity. Then, a time-frequency decomposition with Morlet wavelets is used to find the spectrogram of the component, and the time-varying power is finally computed by averaging the spectrogram in the band 1–45 Hz (7, 16). We will use the name *IC time-varying power* (ICApow) for the final predictor.

#### 2.3.3. EEG Synchronization (PSI and GFS)

Abreu et al. (14) have prompted the idea to focus on the hypersynchronization associated to epileptic events, and quantify it by means of a time-varying metric of coupling between EEG channels. In their paper, they investigate the Phase Slope Index (PSI) and Global Field Synchronization (GFS) and evaluate their performance vs. that of Un and ICApow predictors. By comparing the obtained statistical maps to electrical source imaging maps of the delineated IEDs, they concluded that the PSI metric, computed in a narrow band between 3 and 10 Hz, yielded the best correspondence. Hence, in this paper, we will employ this metric for comparison. The PSI is computed as in Abreu et al. (14) and Mizuhara et al. (18) between a particular pair of EEG channels. Firstly, the PROJIC algorithm is applied, followed by a K-means clustering to detect a set of IED-related ICs, that are used to reconstruct the data while leaving out non-IED-related ICs. The PSI is then computed between every pair of channels. Dimensionality reduction is performed by selecting the channel with the maximal amplitude of the average IED, and subsequently choosing a second channel as the one for which the PSI time course with the first channel had maximal variance. This latter time course was then chosen as the PSI predictor. For completeness, we also include the GFS metric in the same frequency band, computed as in Jann et al. (17). The GFS measures, for every time bin, to which degree the signals of all channels in the data are in or out of phase with each other. Contrary to the PSI, which is computed for a specific pair of channels, the GFS is a “global” metric, in the sense that synchronization between all channels equally is considered.

#### 2.3.4. Time-Varying Power of Enhanced EEG (MWFpow): A New Predictor

We propose to use the available information, in the form of annotated IEDs, to enhance the EEG signal prior to extracting a predictor. Ideally, we would like to retain all the relevant, IED-like activity in the EEG data, and discard all other waveforms, such as noise, artifacts, and background activity. We approach this task by means of the multi-channel Wiener filter (MWF). The MWF has long been known in audio signal processing to enhance acoustic sources, and has recently also been demonstrated to be a powerful tool for generic artifact removal in EEG signals (15). In the latter paper, the authors have shown the excellent capabilities of the MWF to separate various types of artifacts from the EEG by means of spatio-temporal filtering. In this paper, we will use it in its dual form, i.e., not to remove a particular type of EEG activity, but to enhance it. In the current setting we consider *M*-channel EEG signals **x**[*t*] ∈ ℝ^*M*^ at time *t* [notation as in (15)]:

(4) x[t]=d[t]+n[t] ,

in which **d**[*t*] ∈ ℝ^*M*^ represents the IED-related EEG signals, which are superimposed on the background EEG fluctuations, including artifacts and noise, represented by **n**[*t*] ∈ ℝ^*M*^. In its basic form, the multi-channel Wiener filter acts as a spatial filter **W** that maximizes the objective function

(5) $$\underset{W}{\text{min }}\text{E}\{\|d-W^{T}x{\|}^{2}\}$$

and as such tries to extract **d**[*t*] as well as possible (in mean-squared-error sense) from the observations **x**[*t*]. The solution to this problem is

(6) $$W=R_{xx}^{-1}R_{dd}\;,$$

in which ${\text{R}}_{xx}=\text{E}\left\{\text{x}{\text{x}}^{T}\right\}$ is the covariance matrix of the observed signals, and ${\text{R}}_{dd}=\text{E}\left\{\text{x}{\text{d}}^{T}\right\}$ is the covariance matrix of the desired signals **d** (which are unobservable!). If **d** and **n** can be assumed uncorrelated, then **R**_*xx*_ = **R**_*dd*_ + **R**_*nn*_. By splitting the observations of **x**[*t*] into two subsets—time instants $t\in C_{1}$ that cover the occurrences of IEDs, and time instants $t\in C_{0}$ in which no IEDs occur—the latter matrices can be estimated via

(7) $$\begin{array}{l} {\hat{\text{R}}}_{xx}=\frac{1}{\left|C_{1}\right|}\underset{t\in C_{1}}{\sum }\text{x}\left[t\right]\text{x}{\left[t\right]}^{T} \end{array}$$

(8) $$\begin{array}{l} {\hat{\text{R}}}_{nn}=\frac{1}{\left|C_{0}\right|}\underset{t\in C_{0}}{\sum }\text{x}\left[t\right]\text{x}{\left[t\right]}^{T} \end{array}$$

(9) $$\begin{array}{l} {\hat{\text{R}}}_{dd}={\hat{\text{R}}}_{xx}-{\hat{\text{R}}}_{nn} \end{array}$$

Hence, the multi-channel Wiener filter can be found as $\hat{\text{W}}={\hat{\text{R}}}_{xx}^{-1}{\hat{\text{R}}}_{dd}$ and applied to the observed signals to obtain an estimate of clean IED time courses $\hat{\text{d}}={\hat{\text{W}}}^{T}\text{x}$. The MWF can be extended from a purely spatial filter to a spatiotemporal filter, in which not only samples from other channels are used to improve the signal estimation at a particular channel, but also samples from different time instants. In this case, observations are represented in long vectors $\tilde{\text{x}}\left[t\right]$ that are constructed by stacking lagged versions of the data **x**[*t*] at time *t*:

(10) $$\begin{array}{l} \tilde{\text{x}}\left[t\right]=\left[\begin{array}{c} \text{x}\left[t-\tau \right]\\ \vdots \\ \text{x}\left[t-1\right]\\ \text{x}\left[t\right]\\ \text{x}\left[t+1\right]\\ \vdots \\ \text{x}\left[t+\tau \right] \end{array}\right], \end{array}$$

which is followed by the computation of the covariance matrices and the resulting filter as before. This generalization allows finite-impulse-response (FIR) filtering at every channel, and thereby the exploitation of spectral differences between the IEDs and the background EEG. The extra degrees of freedom are described by the number of lags τ, which can be chosen high if enough training data are available. The crucial ingredients for the MWF are two covariance matrices: one covariance matrix ${\hat{\text{R}}}_{xx}\in {\mathbb{R}}^{\left(2M\tau +M\right)\times \left(2M\tau +M\right)}$ of the signal of interest embedded in background EEG (which can be estimated from the EEG during the IED periods) and one covariance matrix ${\hat{\text{R}}}_{nn}\in {\mathbb{R}}^{\left(2M\tau +M\right)\times \left(2M\tau +M\right)}$ of the background EEG itself (which can be estimated from the data during all other periods). Note that these quantities may be estimated over the full length of the EEG recording, or alternatively, over a portion of the recording only^1^. In the latter case, if IED annotation is performed manually, a neurologist may save substantial time by marking IED and non-IED periods in a smaller part of the signal, if these periods can be considered sufficiently representative for the whole signal (15).

Hence, the MWF will aim to enhance the waveforms in the EEG with the same covariance structure as the signal of interest, while suppressing waveforms with a covariance structure similar to the portion of the EEG signals which were marked as not of interest. The output signals $\hat{\text{d}}\left[t\right]$ will then ideally only reconstruct the waveforms at the times of true IEDs, and contain near-zero values everywhere else. Hence, when computing the time-varying power of these filtered signals, and averaging over all channels, a new predictor of IED occurrence is obtained, which we name *multi-channel Wiener filtered EEG time-varying power* (MWFpow). The advantages of such an approach are that (1) the MWF can find additional IEDs, if they were accidently overlooked during annotation, or were obfuscated by superimposed artifacts, (2) the MWF might correct for erroneous IED annotations, if they indeed follow a different covariance structure, (3) the MWF allows for a continuous representation in the output, differentiating IEDs by amplitude and duration, compared to discrete metrics. At low signal-to-noise ratios, the imperfect estimation of the covariance matrices can lead to “over-subtraction” in (9): while ${\hat{\text{R}}}_{dd}$ is expected to be a proper, positive semi-definite matrix, the subtraction of two other estimated covariances is not guaranteed to preserve this property. In order to ensure the positive semi-definiteness of ${\hat{\text{R}}}_{dd}$, one can simultaneously diagonalize ${\hat{\text{R}}}_{xx}$ and ${\hat{\text{R}}}_{nn}$ and perform the subtraction of the two diagonal matrices **D**_*xx*_ and **D**_*nn*_ in the common basis, setting negative entries to zero:

(11) $$\begin{array}{l} {\hat{\text{R}}}_{xx}=\text{V}{\text{D}}_{xx}{\text{V}}^{T} \end{array}$$

(12) $$\begin{array}{l} {\hat{\text{R}}}_{nn}=\text{V}{\text{D}}_{nn}{\text{V}}^{T} \end{array}$$

(13) $$\begin{array}{l} {\left[{\hat{\text{D}}}_{\Delta }\right]}_{ii}=\text{max}\left\{{\left[{\text{D}}_{xx}\right]}_{ii}-{\left[{\text{D}}_{nn}\right]}_{ii},0\right\},\forall i=1\ldots \left(2M\tau +M\right) \end{array}$$

(14) $$\begin{array}{l} {\hat{\text{R}}}_{dd}=\text{V}{\text{D}}_{\Delta }{\text{V}}^{T} \end{array}$$

In (13), [**D**]_*ii*_ denotes the element at position (*i, i*) of a matrix **D**, i.e., the *i*-th diagonal element of **D**. The simultaneous diagonalization in (7) and (8) can be accomplished via a generalized eigenvalue decomposition (GEVD) of matrices ${\hat{\text{R}}}_{xx}$ and ${\hat{\text{R}}}_{nn}$. In the remainder of the paper, we will use GEVD-MWF, as it leads to robust enhancement (15).

### 2.4. EEG-Correlated fMRI Analysis

To map the network of brain regions involved in IED generation and propagation, we relied on the general linear model (GLM) for fMRI (25). We conduct a separate analysis per patient/predictor pair. After convolving an extracted predictor with a canonical model of the hemodynamic response function, we obtain a regressor time series to model the IED-related contribution to every voxel's BOLD signal. As covariates of no interest, we include the six motion-correction parameters, and the average time series in the white matter and the lateral ventricles (cerebrospinal fluid). If necessary, also boxcar regressors are added at moments of substantial scan-to-scan head movement (larger than 1 mm based on the translation parameters). We then calculated statistical T-maps for every IED-predictor separately, interrogating every voxel for its participation in IED generation or propagation. We hypothesized that predictors offer differential modeling power, which manifests itself as the (in)ability to correctly localize the IOZ due to a (mis)match between the modeled and measured BOLD signal. As in Tousseyn et al. (21), we evaluated performance metrics for different statistical maps using different threshold criteria. More precisely, we applied a transformation of the T-map to Z-scores and used different Z-thresholds, familywise error (FWE) correction, and a constraint on the cluster size *k* for active regions. In Tousseyn et al. (21), we have argued that practitioners are to use the threshold of maximal specificity, i.e., producing the fewest false activations (see 2.6). After evaluating a wide range of thresholds in terms of their specificity and sensitivity in localizing the IOZ, we found that a threshold of *Z* > 3.4, combined with a constraint on the cluster size of *k* > 350 voxels (2.8 cm^3^), yielded the highest sensitivity (62%) under the condition of maximal specificity (100%)^2^. Aside from this “optimal” setting, we also evaluate two thresholds that are common in literature: uncorrected *p* < 0.001, and FWE-corrected *p* < 0.05. Lastly, we evaluate a more conservative threshold, i.e., FWE-corrected *p* < 0.05, combined with a cluster constraint of *k* > 350 voxels. In summary, we examined the model's performance at the following thresholds:

1. *p* < 0.001, uncorrected for multiple comparisons, no constraint on cluster size (*Z* > 3.1, *k* > 0)
2. *p* < 0.05, FWE-corrected, no constraint on cluster size(FWE < 0.05, *k* > 0)
3. *p* < 3.37 × 10^−4^, uncorrected for multiple comparisons, cluster larger than 350 voxels (2.8 cm^3^) (*Z* > 3.4, *k* > 350)
4. *p* < 0.05, FWE-corrected, cluster larger than 350 voxels (2.8 cm^3^) (FWE < 0.05, *k* > 0).

### 2.5. Ictal Onset Zone

We compared the statistical maps with a ground truth image of the IOZ to assess its predictive power, as in Tousseyn et al. (21). For patients that had undergone surgical treatment with a successful outcome (ILAE classification 1–3), the manually delineated resection zone was considered as IOZ. In other patients (who were ineligible for or refused surgery), the hypothetical resection zone, based on concordant evidence from multiple modalities other than EEG-fMRI was taken as the best possible estimate of the IOZ.

### 2.6. Model Performance

To quantitatively assess the performance of the models constructed from different IED predictors, we evaluate sensitivity and specificity. Sensitivity is defined as the fraction of “true positives,” i.e., the percentage of patients for which the thresholded statistical map, obtained for a certain model and threshold, overlaps with the IOZ (20). Following the reasoning in Tousseyn et al. (21), we do not consider significantly active voxels or regions outside of the delineated IOZ as false positives. Acknowledging epilepsy as a network disorder, such active regions might reflect seizure or IED propagation, despite not being involved in their generation. For this reason, we use the fMRI data from healthy control subjects to calculate the specificity, i.e., the ratio of false positives. Re-using the IED-predictor from the matched patients, we computed T-maps for the twelve control subjects, and considered control maps with significantly active regions as false positive cases. Analogously, true negative cases are healthy controls for which the statistical map had no suprathreshold regions, and false negative cases were those patients for which no overlap with the IOZ was found. To further distinguish the performance obtained with different IED predictors, we also inspected the cumulative statistical evidence that they produced: we summarized the *T-evidence* as the average *T*-value in a predefined set of voxels. A high value for the T-evidence within the IOZ is then an indication of a good temporal model of the BOLD signal, which is capable of distinguishing IOZ voxels. Note that this metric allows us to rank the performance of different regressors that correctly infer the IOZ (i.e., produce suprathreshold voxels in the IOZ), but even allows to rank the performance of non-sensitive regressors. Hence even when regressors do not produce suprathreshold voxels in the IOZ, they can still be more useful than others if they manage to lift the statistical values, and hence “hint” toward certain regions. Lastly, we tracked the sensitivity when only considering the cluster containing the maximal *T*-value. Following the “network disorder” argumentation, we prefer such a metric over other existing metrics such as the Dice coefficient, since these penalize active voxels out of the IOZ, perhaps wrongfully.

## 3. Results for Patients and Matched Controls

### 3.1. Multi-Channel Wiener Filtering Successfully Enhances IEDs

**Figure 1:**
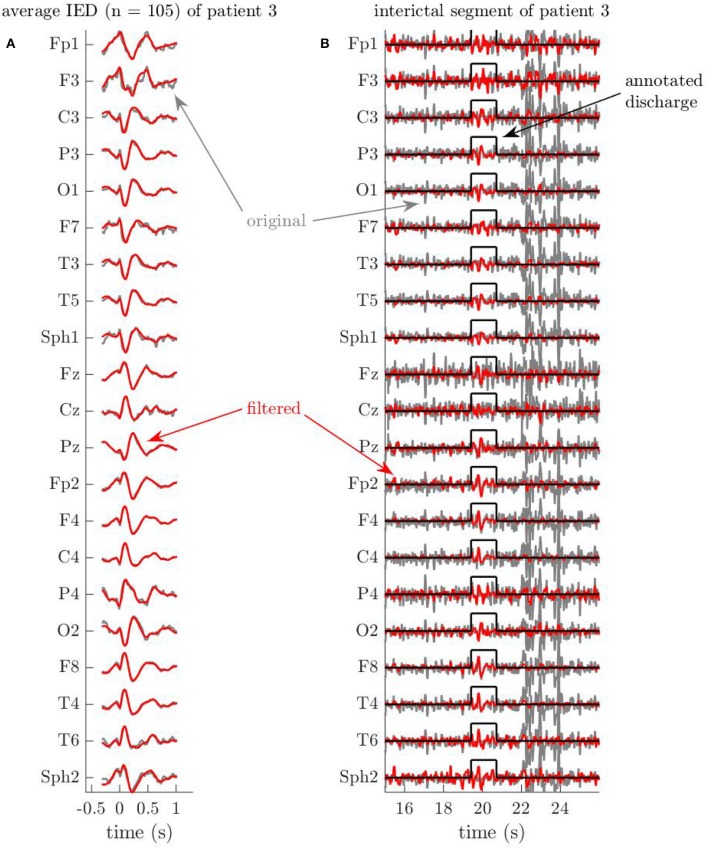
Example EEG segments of patient 3 illustrate the artifact removal capabilities of the Multi-channel Wiener filter (MWF). Time is indicated in seconds relative to the first spike of the discharge. **(A)** Average of all (105) interictal discharges of patient 3, before and after applying the MWF. The MWF properly preserves the signal during annotated periods of interictal discharges, which is evidenced by the strong similarity between the average of the 105 original discharges, and the average of their filtered versions. **(B)** The MWF suppresses a large artifact, while preserving the annotated discharge. The MWF successfully attenuates the artifact, which occurs between 22 and 26 s, because the waveforms do not follow the spatio-temporal statistics of the IEDs, which were used to calibrate the MWF. At the same time, the signals during the discharge around 20 s, which was annotated by the neurologist, stay preserved in the MWF output (compare to the average discharge in A).

We illustrate that the MWF succeeds in enhancing the IEDs in the EEG, while suppressing irrelevant (background) EEG activity. In Figure 1A, we show the average waveform in the annotated IED segments of patient 3, before and after applying the MWF. The excellent correspondence of these two sets of signals on all EEG channels confirms that the MWF leaves—to a large extent—the IEDs intact, respecting the a priori information that was injected by means of the neurologist's annotations. On the other hand, artifacts, such as the one shown in Figure 1B around 22…26 s, are strongly attenuated in the output of the MWF. This example demonstrates that EEG activity that does not follow the spatiotemporal covariance structure ${\hat{\text{R}}}_{dd}$ of the IEDs, that was used to calibrate the MWF, is suppressed in the output of the MWF, as desired. Hence, the MWF performs *signal enhancement* (of the annotated IEDs) and *noise reduction*^3^, improving the signal-to-noise ratio of IEDs.

**Figure 2:**
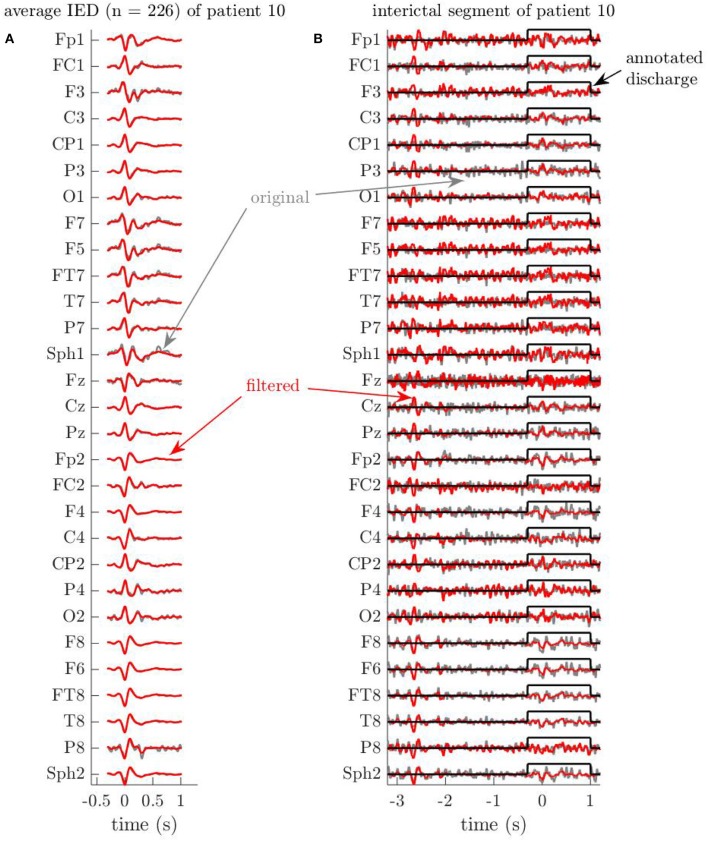
Example EEG segments of patient 10 showcase the error-correcting capabilities of the Multi-channel Wiener filter (MWF). Time is indicated in seconds relative to the first spike of the discharge. **(A)** Average of all (226) interictal discharges of patient 10, before and after applying the MWF. Just as in Figure 1, the MWF preserves the signal during annotated periods of interictal discharges, respecting to large extent the annotations that were made by the human annotator. **(B)** The MWF attenuates an annotated discharge, and finds a new discharge in an unannotated region. Sometimes, annotated discharges are (partially) attenuated, if the corresponding waveforms do not correspond well to the average spatiotemporal statistics that have been used to train the MWF. In this case, this effect is visible in the window of 1.5 s around an annotated discharge, on channels O2, C4, T7, … On the other hand, discharges that were missed by the human annotator may be uncovered by the MWF by enhancing waveforms in the output. In this example, the signal is enhanced between −3 and −2 s relative to the annotated spike. Indeed, the waveforms in this period show striking correspondences to the average of the annotated discharges of this patient in A.

As as second example, we show in Figure 2 excerpts of the EEG signals of patient 10, before and after filtering. In Figure 2A, it is again clear that the MWF appropriately reconstructs the waveforms of most annotated IED segments. Secondly, we indicate another effect of applying the MWF in Figure 2B. The annotated IED in this portion of the EEG corresponds only partially to the average IED shown in Figure 2A, and hence the signal during the annotation period is also attenuated on several channels. While the waveforms on other channels are preserved when they display a higher similarity to the average IED, this shows that a mild errors during annotation may be rectified by the MWF. E.g., a neurologist may mistakenly annotate some dubious EEG segments as IEDs, while they are not. When these “false positive” IEDs are sufficiently limited in number, they do not contaminate the covariance ${\hat{\text{R}}}_{xx}$ in (7) significantly, and can probably be suppressed sufficiently by the MWF. At the same time, “false negative” annotations are conceivable, i.e., when the neurologist overlooks one or more IEDs when inspecting the EEG. The resulting absence of such IEDs in e.g., the Un predictor may lead to an underfitting of the BOLD signal and subsequently to a decreased sensitivity when mapping the ictal onset zone. In Figure 2B we identify such a case. Near the beginning of the shown segment, between −3 and −2 s relative to the annotated IED, the MWF output signal is enhanced on nearly all channels, and waveforms which are very similar to those of the average IED can be discerned. This strongly suggests that this segment contains a true IED, which was missed during annotation, but was recognized by the MWF to follow the same spatiotemporal signal characteristics as the other annotated IEDs. Several such segments were found for this patient, and we note that the MWFpow predictor was sensitive for this patient, contrary to the Un predictor (see infra), which reinforces the hypothesis that the MWF indeed succeeds in “discovering” previously undetected IEDs. Hence, the MWF also has *error-correcting capabilities*.

For all data, we assigned all samples within a window of 0.5 s before until 1.0 s after the first peak of any IED to $C_{1}$, and subsequently used GEVD-MWF as described by Equations (11)–(14). We chose τ = 4 (corresponding to lags between −16 and +16 ms, in increments of 4 ms), since we observed no significant changes to the MWF output signals for higher values of the parameter (i.e., the output signal-to-noise ratio saturates) and wanted to ensure well-conditioned covariance estimation.

### 3.2. Sensitivity and Specificity of Different Predictors

**Figure 3:**
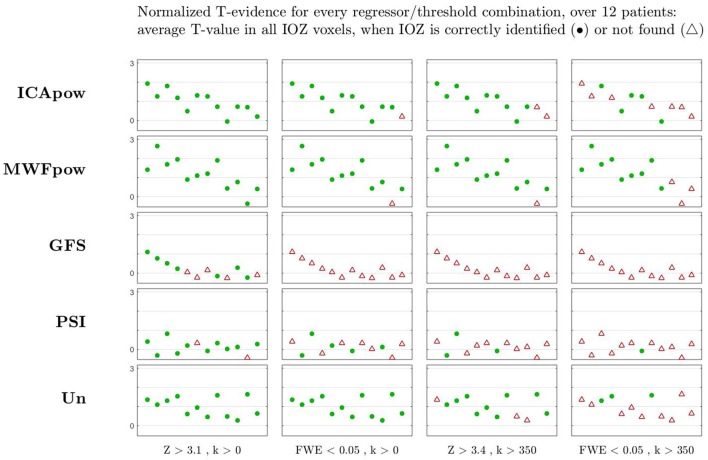
The statistical evidence found in the ictal onset zone is highest for especially the MWFpow and also ICApow regressors, and lower for the other regressor types, for most patients (shown by individual markers). This is correlated with a higher sensitivity (i.e., more true positives) for these two regressors, especially at higher (more selective) thresholds to the right of the figure, where synchronization regressors (PSI and GFS) and the unitary regressor Un fail. At different thresholds, cases with clusters of suprathreshold *T*-values that overlap with the ictal onset zone are counted as true positives  (•), other cases are considered false negatives  (△). At the lowest threshold (*Z* > 3.1, *k* > 0), all regressors were 0% specific (i.e., produced suprathreshold activations in all healthy controls), and at the three higher thresholds, all of them were 100% specific (no suprathreshold activations in healthy controls), except for one false positive case produced by ICApow at FWE-corrected *p* < 0.05, *k* > 0.

We found that the choice of predictor largely influences the sensitivity of IOZ detection. Figure 3 supports this observation by highlighting true positive detections with filled green markers, and false negative detections with empty red markers, at various combinations of predictor and statistical threshold. To compare different predictors beyond their binary detection performance, we plot the T-evidence within the IOZ^4^ for every patient (cfr. section 2.6). Note that the specificity cannot be determined from the figure, but followed a simple pattern in our results: at the lowest threshold, all predictors were 0% specific, and at the three higher thresholds, all of them were 100% specific, except for one false positive case produced by ICApow at FWE-corrected *p* < 0.05.

At the “optimal” [according to (21)] threshold of *Z* > 3.4 with cluster size constraint, the MWFpow predictor achieves the highest sensitivity (92%), outperforming ICApow (83%), Un (75%), PSI (25%) and GFS (0%). At this significance level, all of the predictors led to a specificity of 100%. At FWE-corrected *p* < 0.05, the Un predictor is 100% sensitive, better than the MWFpow and ICApow predictors. At this threshold however, the latter produced one false positive (92% specific, not shown in figure). At the lowest significance level, the sensitivity of most predictors (except GFS) is very good, yet all are absolutely non-specific (0%). At the other side of the spectrum, the MWFpow predictor was most robust over thresholds, still correctly detecting 75% of IOZs at the highest threshold, whereas the sensitivity of other predictors decreased to 42% and lower.

These results prove the viability of MWFpow as a new model in EEG-correlated fMRI analysis for (refractory) epilepsy. In general, the two synchronization metrics performed poorly: for no threshold did GFS reach an acceptable balance of sensitivity/specificity, and PSI was not sensitive in general. We noticed that the case which was wrongfully delineated by MWFpow at the three highest thresholds (the 11^th^ in the figure), corresponded to a data recording in which particularly few (6) IEDs were observed, compared to more than hundred IEDs in many other recordings. We hypothesize that the low number of IEDs resulted in a “data deficiency” when training the MWF: with few data segments from one class, it is likely that a good spatio-temporal filter cannot be reliably determined. We investigated the filtered EEG data of this recording and found indeed several remaining, high-amplitude artifacts in the data. After manually masking out these artifact periods (by setting the samples to zero), the resulting MWFpow predictor was sensitive for this case as well (not shown). We therefore advocate for a “quick and dirty” inspection of high-amplitude periods in the filtered EEG, in order to manually correct artifact segments where needed, especially for datasets with few IEDs.

Besides binary metrics (successful or unsuccessful localization of the IOZ), we also point out the difference in T-evidence among different predictors. The MWFpow predictor is capable of identifying the highest statistical evidence in the ictal onset zone (IOZ), for many patients, as supported by Figure 3. The Un and ICApow predictors produce less evidence, and the synchronization metrics underperform with low evidence levels. This corroborates the higher sensitivity of the MWFpow predictor (and also the ICApow predictor) for interictal events, especially at higher (more selective) thresholds, where synchronization regressors (PSI and GFS) fail. Hence, the T-evidence metric confirms that the MWFpow predictor is a robust predictor which allows to find the IOZ with higher statistical significance than the other predictors.

**Figure 4:**
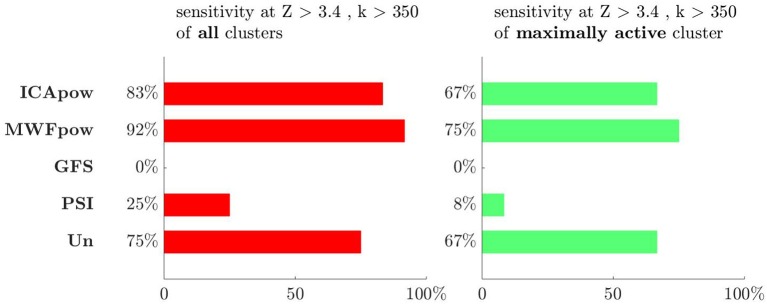
The sensitivity is highest for the MWFpow regressor, whether the overlap with the ictal onset zone of all clusters (left) or only of the maximally active cluster (right) is considered. At the threshold which was found to be most selective in Tousseyn et al. (21), i.e., *Z* > 3.4 with cluster size constraint *k* > 350 voxels or 2.8 cm^3^, the ranking of all regressors is the same for both types of sensitivity. The colors for both types of sensitivity correspond to the colors of the clusters in Figures 5–7.

To further investigate the diagnostic relevance of the different regressors, we considered the distribution of activation clusters in the brain. We therefore evaluated the sensitivity in two different ways: (1) by counting a true positive when any activation cluster overlaps with the IOZ or (2) by only counting a true positive when the cluster containing the voxel with the highest *T*-value overlaps with the IOZ. Evidently, the sensitivity of the maximally active cluster is always upper-bounded by the sensitivity of all clusters. We show these two types of sensitivity, evaluated at the threshold of *Z* > 3.4 with cluster size constraint *k* > 2.8 cm^3^, in Figure 4. For the alternative type of sensitivity, a similar pattern as before emerges: the performance of the MWFpow is still the highest among all regressors. The interpretation of this figure is that a reader would be able to correctly identify the IOZ in 75% of cases by picking the maximally active cluster after EEG-correlated fMRI analysis using the MWFpow regressors, and with a lower success rate for other types of regressors.

### 3.3. Activation Maps of Individual Patients

As a final part of our analysis, we inspect the statistical maps at *Z* > 3.4 with cluster size constraint *k* > 350 voxels (2.8 cm^3^) for some interesting individual cases. For visualization purposes, we show thresholded maps, though we stress that T-evidences were computed from the unthresholded *T*-values.

**Figure 5:**
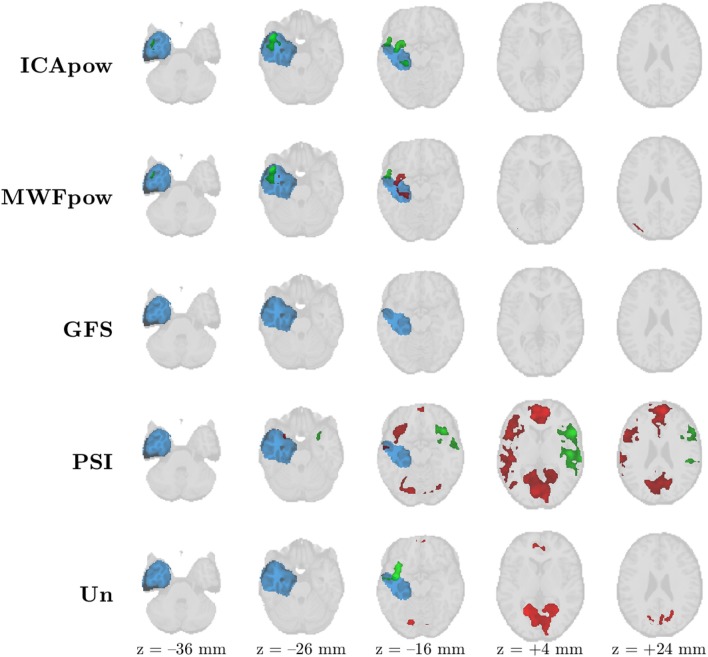
Patient 7's ictal onset zone (blue) was correctly detected by the EEG-correlated fMRI activation maps (red) of all predictors except GFS at *Z* > 3.4 with cluster size constraint *k* > 350 voxels (2.8 cm^3^). The maps of MWFpow and ICApow are remarkably similar, while large differences with the other predictors exist. The maximally active cluster for every predictor is marked in green (instead of red).

Firstly, we inspect the maps for the 7^th^ patient, in Figure 5. As can be concluded from Figure 3, all predictors except for GFS produced a correct detection of the IOZ. However, large differences in the active clusters exist. The MWFpow and ICApow predictors led to very similar maps, with the maximally active cluster concentrated in and around the IOZ. Also the Un and PSI predictors are sensitive, though only a few suprathreshold voxels overlap with the IOZ, and the maximally active cluster falls out of the IOZ for the latter. Many voxels survive the threshold for the PSI map, but comparison with the maps from the other predictors leads to believe that these are in fact false positive findings. The GFS predictor produced a very low (but non-zero) T-evidence for this patient, but since no cluster of voxels met the threshold on *T*-value and size, no active voxels are shown here.

**Figure 6:**
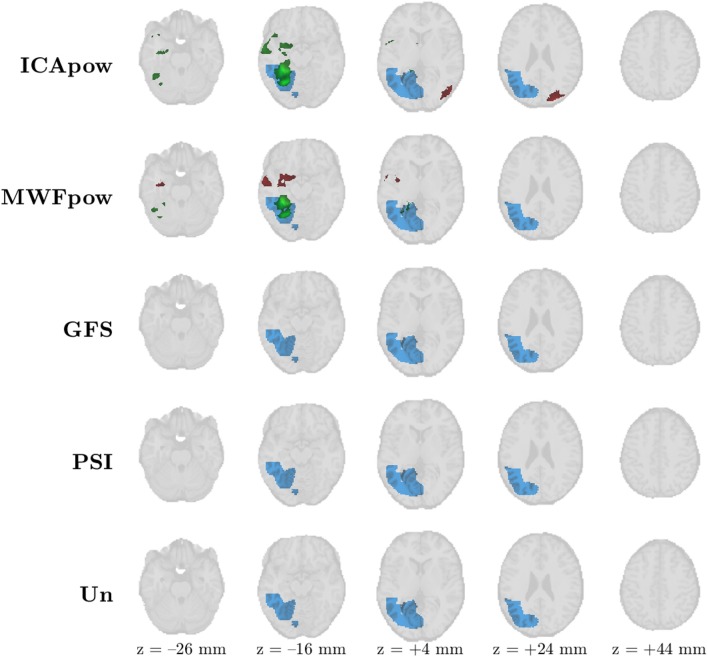
Patient 9's ictal onset zone (blue) was only correctly detected by the EEG-correlated fMRI activation maps (red) of the MWFpow and ICApow predictors at *Z* > 3.4 with cluster size constraint *k* > 350 voxels (2.8 cm^3^). The ICApow predictor produced one additional active cluster outside of the delineated ictal onset zone. The maximally active cluster for every predictor is marked in green (instead of red).

Secondly, we show in Figure 6 the maps of the 9^th^ patient, a “difficult case” for which the T-evidence was low, irrespective of the predictor. Only the ICApow and MWFpow predictors led to maps with clusters that met the constraints. For both predictors, the maximally active cluster correctly indicates the IOZ. In both cases, there is also a second cluster that falls outside the IOZ. The maximally active cluster of the ICApow predictor corresponds largely to the two most active clusters of the MWFpow predictor, where it seems that this cluster has been “broken” into two constituent parts. None of the other predictors led to suprathreshold clusters, illustrating the need for tailored, data-driven, and robust predictors such as MWFpow and ICApow.

**Figure 7:**
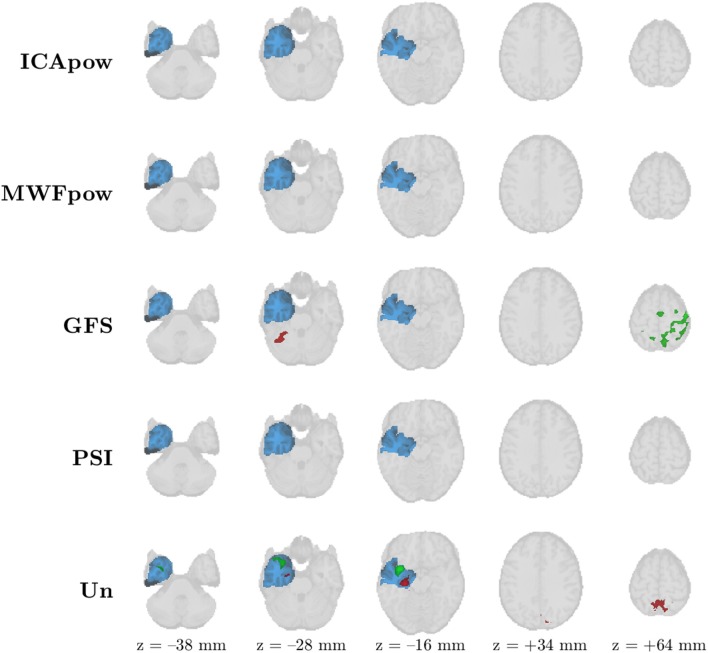
Patient 11's ictal onset zone (blue) was only detected by the EEG-correlated fMRI activation map (red) of the Un predictor at *Z* > 3.4 with cluster size constraint *k* > 350 voxels (2.8 cm^3^). The sparse occurrence of IEDs during the recording may have precluded proper tuning of the MWFpow predictor, causing it to miss the ictal onset zone. The maximally active cluster for every predictor is marked in green (instead of red).

Lastly, we show an atypical case in Figure 7, corresponding to the 11^th^ patient of our analysis. From Figure 3 we recall that correct detection of the IOZ was only attained for the Un predictor. Indeed, nearly all activation maps are empty, besides three distinct active clusters in the Un map, two of which overlap the IOZ, and surprisingly also two active clusters in the GFS map, outside of the IOZ. As argued earlier, we hypothesize that the limited number of interictal spike examples (6) precluded the estimation of a robust, data-driven IED representation. The extremely simple, signal-independent Un predictor may be more robust in such cases. If this hypothesis is correct, we consider this as a “foreseeable failure,” of the data-driven methods. I.e., for analyses where the recording contains merely a few interictal discharges, one may take additional action: (1) the practitioner may still rely on data-driven predictors (such as MWFpow), given that (s)he still manually inspects and potentially corrects leftover artifacts in the predictor, as we have done, or (2) rely on predictors such as Un which do not have to “learn” from data examples. Despite this caution, we note that the MWFpow predictor did allow a correct detection for two other patients with few observed IEDs (patients 1 and 8 had 15 and 11 IEDs, respectively), even at the most stringent threshold.

## 4. Discussion

We have examined the modeling power of different EEG-derived predictors to map the ictal onset zone in patients with refractory epilepsy through EEG-correlated fMRI analysis. We compared existing predictors, which are based on either unitary representation of IEDs (Un), on time-varying power of estimated IED activity (ICApow) or on local (PSI) or global (GFS) neural synchronization, and proposed a new predictor based on supervised IED enhancement (MWFpow). Through a rigorous analysis of sensitivity and specificity at various thresholds, we have demonstrated the usefulness of the (MWFpow) predictor when mapping the epileptic network, and more in particular the IOZ. By extracting only the relevant, IED-related information from the EEG, the new method allows for successful localization of the IOZ in a higher fraction of cases, and with higher statistical evidence.

The idea to separate one “source of interest” from multivariate data with multiple active sources is not unique to the MWF methodology. As a very popular tool in EEG processing, ICA essentially has the same objective and is even more ambitious, because multiple sources are estimated. Both ICA and the MWF are types of spatial filters (note that we have indicated this with the same symbol $\hat{\text{W}}$ in section 2) that have been used for EEG signal enhancement. The crucial distinction is that ICA is completely unsupervised (i.e., requires no user input), whereas the MWF is supervised and leverages prior knowledge in the form of annotated time segments in (a portion of) the data^5^. In the current study, both approaches have been compared, as the ICApow regressors are derived after estimating independent components (ICs) and identifying the IC that is most related to IEDs.

The success of the MWFpow predictor can be attributed to several factors. First of all, the new predictor is a continuous IED representation, in the sense that individual variations in IED amplitude and duration are preserved, as opposed to the discrete nature of the very popular Un predictor. Furthermore, the MWFpow predictor finds a new balance between supervised (user-driven) and unsupervised (data-driven) operation. On the former end of the spectrum resides the Un predictor, which strictly models only those IEDs which have been marked by a neurologist. As such, this model may fail to capture relevant IED-specific variations, and does not treat IED-like events that have been missed (or wrongfully annotated) by the user. Toward the latter side of the spectrum is the ICApow predictor, which extracts an IED representation by decomposing the data in several ICs and only withholding the IC that corresponds most strongly to the user-annotated IEDs. We have shown that the MWF paradigm allows in fact a correction of the annotated IEDs: based on the estimated covariance structure, new IED-like events may be found in the MWF outputs if they match the learned characteristical spatiotemporal correlations. It may seem as though there is a risk for “positive feedback” here, since initially undetected IED events will contribute to the noise covariance ${\hat{\text{R}}}_{nn}$ and as such belong to the class of waveforms the MWF will suppress. Luckily however, IEDs are sparse events, and the fraction of truly non-IED EEG is usually far higher than that of (undetected) IED segments. Thus, ${\hat{\text{R}}}_{nn}$ will only be marginally impacted by the leakage of these undetected events. If a sufficiently representative covariance ${\hat{\text{R}}}_{dd}$ of the desired signals has been estimated, the MWF may then recover these undetected IEDs from the data. Contrary to the effect on ${\hat{\text{R}}}_{nn}$, the additional reconstruction of these events in the MWF outputs *is* significant, precisely because of the sparse nature of the IEDs.

Our results contrast those of Abreu et al. (14), where the PSI predictor was found to outperform ICApow, GFS and Un. In our study, we have found an opposite trend, namely that the PSI predictor is considerable less sensitive than the ICApow and especially the MWFpow predictor. Several arguments might explain this result. Notably, only four patients with observed IEDs during EEG-fMRI recording were included in the analysis in Abreu et al. (14). In our study, we investigated a considerably larger sample of twelve patients, and hence we suspect that our results have more generalization power. Furthermore, assessment of the BOLD activation maps in Abreu et al. (14) was performed by checking correspondence with electrical source imaging maps. However, since the EEG was only recorded at 31 electrodes, the resulting source maps may contain substantial localization errors (26–28) and may not serve as a reliable ground truth to evaluate “correctness” of found active BOLD clusters. Instead, we have opted for another way to evaluate sensitivity and specificity, which is based on the anatomical mapping of the ictal onset zone directly. In the patients that had a successful outcome following surgery, the ictal onset zone has been defined as that part of the cortex which had been removed or resected. Consequently, this definition of IOZ certainly encompassed the epileptogenic zone, as has been described in section 2. For patients that had not become seizure-free, we used evidence from other modalities such as SPECT and video-EEG, to make a best guess about the delineation of the IOZ. Besides the differences in validation and patient cohort, we have tried to replicate the processing pipeline of Abreu et al. (14) as closely as possible, to allow fair comparison. However, small differential effects due to scanner specifics or preprocessing of the raw EEG data may still persist.

Two limitations of the study need to be underlined. Since nine out of twelve studied patients had temporal lobe epilepsy, we can not yet conclude that the new method generalizes well to other types of epilepsy, and further studies are advocated to investigate this. Furthermore, for many patients, relatively many IED annotations were available (see Table 2). It is to be expected that the sensitivity of the proposed MWFpow predictor (and also that of all other predictors) on average degrades when only few IEDs occur during the fMRI recording, as is the case for patient 11. Nonetheless, a low number of observed IEDs did not preclude correct IOZ detection in two other cases (patients 1 and 8). This is somewhat encouraging: the method's performance does not collapse entirely, but rather offers a reasonable robustness, under “‘IED-scarce conditions”. In some cases, no IEDs are observed on the EEG recorded during the fMRI session. While this precludes the use of the Un predictor, it is still possible to use MWFpow predictor, if an additional EEG recording of the patient (out of the scanner) is available in which IEDs occur. In such cases, the MWF can be “calibrated” on this set of EEG signals, and then used to filter the EEG signals recorded during the fMRI session. As shown in Grouiller et al. (29) using an analogous method based on correlation with an IED template, such an approach can still produce very meaningful spatial maps. A similar approach may be followed for the ICApow and PSI predictors (for which also at least one observed IED is needed), while for the GFS predictor, no IED information is used (which may be one reason for its weak performance in this study).

In summary, we advocate for the use of the newly developed MWFpow predictor, when mapping interictal brain activity using EEG-correlated fMRI. This method works in an almost fully data-driven fashion, and uses examples of IEDs as a main ingredient to enhance the EEG signals and obtain a robust representation of the interictal activity to subsequently model the BOLD signal. The minimal user input that is required consists of some annotations of IEDs in recorded EEG data of a patient. However, a neurologist or EEG reader may be assisted for this by several detection methods, such as described in Tousseyn et al. (22), Nonclercq et al. (30), Grouiller et al. (29), and Oikonomou et al. (31). When labeling IEDs manually, time can be saved by annotating only a representative portion of the EEG recording. Especially for patients with many observed IEDs, the MWFpow predictor has been shown to produce very sensitive and yet maximally selective results, and may therefore be a valuable tool in the presurgical work-up of refractory epilepsy.

## Data Availability

The datasets for this study will not be made publicly available because the patients and healthy participants in the study have not given their approval for the data to be made public.

## Ethics Statement

This study was carried out in accordance with the recommendations of the International Conference on Harmonization guidelines on Good Clinical Practice with written informed consent from all subjects. All subjects gave written informed consent in accordance with the Declaration of Helsinki. The protocol was approved by the Medical Ethics Committee of the University Hospitals KU Leuven.

## Author Contributions

SVE, BH, PD, WV, and SVH designed the experiments and wrote and revised the manuscript. WV and PD collected the data for the experiments. SVE carried out all experiments.

### Conflict of Interest Statement

The authors declare that the research was conducted in the absence of any commercial or financial relationships that could be construed as a potential conflict of interest.

## Footnotes

^1^Since the inverse of ${\hat{\text{R}}}_{xx}$ is used in the computation of the filter in (5), sufficiently many EEG segments should be marked to ensure that this matrix is full-rank and hence invertible. Conversely, ${\hat{\text{R}}}_{dd}$ may be rank-deficient, e.g., in case of very stationary IEDs (15).

^2^Under the current interpretation of the performance metrics, a predictor may be made arbitrarily sensitive by lowering the threshold appropriately, thereby (often dramatically) sacrificing specificity.

^3^In this context, with the term “noise,” we mean all waveforms or activity in the EEG that are of no interest to the task of mapping the ictal onset zone. This includes various types of artifacts, oscillatory activity, interference, which are unrelated to interictal epileptiform activity. Conversely, all IEDs jointly constitute the “signal.”

^4^The T-evidence computes the average value of unthresholded T-scores in the IOZ, and as such is independent of the imposed threshold.

^5^In the construction of the ICApow predictor, the most IED-like independent component is selected in a supervised fashion, namely based on similarity with user-annotated IEDs. However, the user input has no influence on the enhancement itself, which is the decomposition into independent components.
